# Supplementary material for: A barley pan-transcriptome reveals layers of genotype-dependent transcriptional complexity
Source: Nat Genet. 2025 Feb 3;57(2):441–50. doi: 10.1038/s41588-024-02069-y (PMC11821519; doi:10.1038/s41588-024-02069-y)
Supplement: Supplementary file 1 — Reporting Summary [file 41588_2024_2069_MOESM1_ESM.pdf]

Corresponding author(s): Robbie Waugh, Runxuan Zhang, Craig Simpson and Micha Bayer

Last updated by author(s): Nov 4, 2024

Reporting Summary

Nature Portfolio wishes to improve the reproducibility of the work that we publish. This form provides structure for consistency and transparency in reporting. For further information on Nature Portfolio policies, see our Editorial Policies and the Editorial Policy Checklist.

Statistics

For all statistical analyses, confirm that the following items are present in the figure legend, table legend, main text, or Methods section.

n/a	Confirmed	
☐	☒	The exact sample size (*n*) for each experimental group/condition, given as a discrete number and unit of measurement
☐	☒	A statement on whether measurements were taken from distinct samples or whether the same sample was measured repeatedly
☐	☒	The statistical test(s) used AND whether they are one- or two-sided<br>*Only common tests should be described solely by name; describe more complex techniques in the Methods section.*
☐	☒	A description of all covariates tested
☐	☒	A description of any assumptions or corrections, such as tests of normality and adjustment for multiple comparisons
☐	☒	A full description of the statistical parameters including central tendency (e.g. means) or other basic estimates (e.g. regression coefficient) AND variation (e.g. standard deviation) or associated estimates of uncertainty (e.g. confidence intervals)
☐	☒	For null hypothesis testing, the test statistic (e.g. *F*, *t*, *r*) with confidence intervals, effect sizes, degrees of freedom and *P* value noted<br>*Give P values as exact values whenever suitable.*
☒	☐	For Bayesian analysis, information on the choice of priors and Markov chain Monte Carlo settings
☒	☐	For hierarchical and complex designs, identification of the appropriate level for tests and full reporting of outcomes
☒	☐	Estimates of effect sizes (e.g. Cohen's *d*, Pearson's *r*), indicating how they were calculated

Our web collection on statistics for biologists contains articles on many of the points above.

Software and code

Policy information about availability of computer code

Data collection: No software were used for data collection.

Data analysis: Multiple published software packages were used in the analyses and are described at https://github.com/cropgeeks/barleyPantranscriptome. Specific customized scripts (e.g. for gene cluster filtering) are included in a common folder at https://github.com/cropgeeks/barleyPantranscriptome/tree/main/scripts. Tools and software was used in the data analysis include: Fastp v0.20.1, STAR v2.7.8a, Stringtie v2.1.5, Scallop v0.10.5, IsoSeqv3 v3.4.0, TAMA (Dec 24th, 2022), PSVCP v1.0.1, Minimap2 v2.24, Transuite v0.2.2, InterProScan v5.59-91.0, Salmon v1.4.0, 3D RNA-seq v2.0.1, CD-HIT v4.8.1 , BLAST 2.15.0, SyRI v1.6.3, plotsr v0.5.4, Polyester v1.29.1, DESeq2 v1.34.0, WGCNA v1.69, Mercator4 v5.0, clusterProfiler v4.6, Python v3.10.12, networkx v3.1, Netgraph v4.12.11, OrthoFinder v2.5.5, RTDmaker v0.1.5, OmicsBox v3.1.2 and seqPattern v1.32

For manuscripts utilizing custom algorithms or software that are central to the research but not yet described in published literature, software must be made available to editors and reviewers. We strongly encourage code deposition in a community repository (e.g. GitHub). See the Nature Portfolio guidelines for submitting code & software for further information.

Data

Policy information about availability of data

All manuscripts must include a data availability statement. This statement should provide the following information, where applicable:

- Accession codes, unique identifiers, or web links for publicly available datasets
- A description of any restrictions on data availability
- For clinical datasets or third party data, please ensure that the statement adheres to our policy

All raw data is available through the European Nucleotide Archive (https://www.ebi.ac.uk/ena/browser/home). Raw Illumina reads are available as BioProject accession PRJEB64639 (https://www.ebi.ac.uk/ena/browser/view/PRJEB64639), Iso-Seq CCS reads are available as BioProject accession PRJEB64637 (https://www.ebi.ac.uk/ena/browser/view/PRJEB64637). All data underpinning PanBaRT20 and MorexGeneAtlas are also available in Eorna (https://ics.hutton.ac.uk/panbart20/index.html) and (https://ics.hutton.ac.uk/morexgeneatlas/index.html). The Morex V3 pseudomolecules are available at http://doi.org/10.5447/ipk/2021/3. All supplementary data files have been uploaded to Figshare repositories and can be accessed here: https://figshare.com/s/1a6d27f41385efe5a790?file=47048692, https://figshare.com/s/1a6d27f41385efe5a790?file=47048698 https://figshare.com/s/1a6d27f41385efe5a790?file=47048704

Research involving human participants, their data, or biological material

Policy information about studies with human participants or human data. See also policy information about sex, gender (identity/presentation), and sexual orientation and race, ethnicity and racism.

Reporting on sex and gender	n/a
Reporting on race, ethnicity, or other socially relevant groupings	n/a
Population characteristics	n/a
Recruitment	n/a
Ethics oversight	n/a

Note that full information on the approval of the study protocol must also be provided in the manuscript.

Field-specific reporting

Please select the one below that is the best fit for your research. If you are not sure, read the appropriate sections before making your selection.

☒ Life sciences ☐ Behavioural & social sciences ☐ Ecological, evolutionary & environmental sciences

For a reference copy of the document with all sections, see nature.com/documents/nr-reporting-summary-flat.pdf

Life sciences study design

All studies must disclose on these points even when the disclosure is negative.

Sample size	The 20 barley pan-genome genotypes8 were used to prepare a barley pan-transcriptome. These are Akashinriki, Barke, ZDM02064, ZDM01467, B1K-04-12, GoldenPromise, Hockett, HOR10350, HOR13821, HOR13942, HOR21599, HOR3081, HOR3365, HOR7552, HOR8148, HOR9043, Igri, Morex, OUN333 and RGT Planet. Five different tissue/organ types including embryo, mesocotyl and seminal roots (together referred to throughout as embryonic tissue), seedling shoot, seedling root, inflorescence and caryopsis were collected in three biological replicates amounting to 300 samples.
Data exclusions	Pan Transcriptome RNA-seq data: 2 samples (HOR10350 Caryopsis rep3 and HOR7552 Root rep2) were excluded due to poor RNA quality. Hor8148_In3 was identified as mislabelled thus also being excluded.<br>CNV analyses: Gene clusters with a median expression below 10 TPM were excluded from association analyses between CNV and expression.<br>Gene network analyses: Out of 13,680 orthologous single copy genes, 13,652 genes passed the filter of lowly expressed genes (TPM > 0.5 in minimum 2 biological replicates) in all accessions and were propagated to the following analysis. From the original 297 samples, principal component analysis (PCA) revealed that one sample (ZDM01467_In2) was a severe outlier, therefore, it was removed from further network analysis.<br>For differential expression analysis from the Morex atlas: Transcripts determined as expressed in ≤ 2 of the samples with count per million reads (CPM) ≤ 10 were removed from further analysis.<br>All data exclusions are described in the manuscript 'Main Text' and/or 'Methods'
Replication	Three biological replicates per sample except 2 samples excluded due to poor RNA quality and one sample identified as mislabelled as described above
Randomization	No randomization is required as the plant background and condition is exactly the same. There is no biases on how we choose the plants or tissues.

Blinding	No blinding is required as the there is no participant's biases involved in this plant study

Reporting for specific materials, systems and methods

We require information from authors about some types of materials, experimental systems and methods used in many studies. Here, indicate whether each material, system or method listed is relevant to your study. If you are not sure if a list item applies to your research, read the appropriate section before selecting a response.

Materials & experimental systems

n/a	Involved in the study	
☒	☐	Antibodies
☒	☐	Eukaryotic cell lines
☒	☐	Palaeontology and archaeology
☒	☐	Animals and other organisms
☒	☐	Clinical data
☒	☐	Dual use research of concern
☐	☒	Plants

Methods

n/a	Involved in the study	
☒	☐	ChIP-seq
☒	☐	Flow cytometry
☒	☐	MRI-based neuroimaging

Dual use research of concern

Policy information about dual use research of concern

Hazards

Could the accidental, deliberate or reckless misuse of agents or technologies generated in the work, or the application of information presented in the manuscript, pose a threat to:

No	Yes	
☒	☐	Public health
☒	☐	National security
☒	☐	Crops and/or livestock
☒	☐	Ecosystems
☒	☐	Any other significant area

Experiments of concern

Does the work involve any of these experiments of concern:

No	Yes	
☒	☐	Demonstrate how to render a vaccine ineffective
☒	☐	Confer resistance to therapeutically useful antibiotics or antiviral agents
☒	☐	Enhance the virulence of a pathogen or render a nonpathogen virulent
☒	☐	Increase transmissibility of a pathogen
☒	☐	Alter the host range of a pathogen
☒	☐	Enable evasion of diagnostic/detection modalities
☒	☐	Enable the weaponization of a biological agent or toxin
☒	☐	Any other potentially harmful combination of experiments and agents

Plants

Seed stocks	Inbred seed stocks for the pan-transcriptome study were obtained from IPK-Gatersleben as described in Supplementary Table 2 and Jayakodi, M. et al. (2020 and 2023) and are available from German federal ex situ genebank at IPK Gatersleben.de. For the 'inversion study' seed were obtained from the JHI 'seed store' and are as described in Schreiber, M. et al (2023) https://doi:10.1101/2023.03.06.531259 and listed in Supplementary table 7 and are available from the authors on request.
Novel plant genotypes	n/a
Authentication	Genotypes for pan-transcriptome studies were verified by comparison to SNP polymorphism data derived from the same genotypes used in the barley pan-genome studies of Jayakodi et al. The same inbred seed stocks were used for both studies. For the '141Mb inversion' study all genotypes were verified by comparing RNAseq derived SNPs to barley 50K SNP chip data deposited in a reference 'Germinate' database. For the 'Morex atlas' study all sequences were labeled 'cv. Morex' in the SRA and were assumed to be genuine cv. Morex genotypes.
