# Supplementary material for: A barley pan-transcriptome reveals layers of genotype-dependent transcriptional complexity
Source: Nat Genet. 2025 Feb 3;57(2):441–50. doi: 10.1038/s41588-024-02069-y (PMC11821519; doi:10.1038/s41588-024-02069-y)
Supplement: Supplementary file 3 — Links to three figshare repositories containing Supplementary Data 1–8 and 10–14, Supplementary Data 9 and Supplementary Data 15. [file 41588_2024_2069_MOESM3_ESM.pdf]

Supplementary Data Guo et al NG-A65801-T

‘A barley pan-transcriptome reveals layers of genotype-dependent transcriptional complexity’ by Guo et al

All Supplementary Data associated with this manuscript have been uploaded to Figshare and can be found at the following address:

https://figshare.com/s/083ac85b4199340685c5

1. https://figshare.com/s/083ac85b4199340685c5?file=51219023
   Contains the .zip file titled **Guo et al Supplementary Data1-8_10-14.xlsx** which contains twelve datasets referred to individually in the main text as Supplementary Data files 1-8 and 10-14. They are presented in tabular format as separate sheets in a joint Excel formatted file as follows:.

   Supplementary Data 1: Basic statistics of the RTDs
   Supplementary Data 2: Ordering of genotypes incorporated into the linear pan-genome.
   Supplementary Data 3: Gene categories of GsRTD
   Supplementary Data 4: Alternative splicing events for highly expressed transcripts from core-single-copy genes (average TPM > 10)
   Supplementary Data 5: Gene copy number variation cluster significantly correlated with the gene expression.
   Supplementary Data 6: C-repeat/DRE-Binding Factor (CBF) genes identifier in GsRTD and their location in the genome
   Supplementary Data 7: Genotypes with the 141Mb 7H inversion and non-inversion
   Supplementary Data 8: Differentially expressed genes in the 7H inversion.
   Supplementary Data 10: Detailed example of a split pattern in Golden Promise
   Supplementary Data 11: Barley cv. Morex Expression Atlas Metadata
   Supplementary Data 12: GA-Pathway gene expression in PanTs experiment
   Supplementary Data 13: Yield and agro data for Ga2ox3-7
   Supplementary Data 14: Statistics GA2ox and 7

2. https://figshare.com/s/083ac85b4199340685c5?file=51221972
   Contains the .zip file titled **Guo et al SupplementaryData9.csv** which is a large csv file containing genotype-specific co-expression network results (modules and community assignments) with annotation and MorexV3 gene IDs.

3. https://figshare.com/s/083ac85b4199340685c5?file=51221996
   Contains a large tab delimited text file titled **Guo et al Supplementary Data15.txt** which provides details of how genes/transcripts from the genotype specific RTDs map onto genes in PanBaRT20.
